# Multidrug-Resistant Tuberculosis during Pregnancy: Two Case Reports and Review of the Literature

**DOI:** 10.1155/2016/1536281

**Published:** 2016-02-24

**Authors:** Minakshi Rohilla, Bharti Joshi, Vanita Jain, Jasvinder Kalra, G. R. V. Prasad

**Affiliations:** Department of Obstetrics & Gynaecology, PGIMER, Chandigarh 160012, India

## Abstract

Multidrug-resistant tuberculosis (MDR-TB) is identified from the time of introduction of antituberculosis treatment and is a known worldwide public health crisis affecting women of reproductive age group. Management issues raised by pregnant women with MDR tuberculosis are challenging due to the limited clinical experience available with the use of second line drugs. We hereby report two cases of MDR-TB during pregnancy: one patient was on second line drugs, while another one was evaluated and diagnosed to have MDR-TB in last trimester. At 6 months of follow-up both mothers and babies are doing well. The approach to such cases along with review of the literature is discussed.

## 1. Introduction

Multidrug-resistant TB (MDR-TB) is described as* M. tuberculosis* infection resistant to rifampicin and isoniazid with or without resistance to other drugs. This form of tuberculosis is prevalent in nearly all countries [1]. India had an estimated 63,000 notified cases in 2010, the highest in the Southeast Asia region. The reported incidence of MDR-TB in India is 1–3% in new cases and around 12%–17% in retreatment cases (Tuberculosis Research Center (TRC), National Tuberculosis Institute (NTI), and Revised National Tuberculosis Control Programme (RNTCP) [2–4]). So far, there are less than 100 reported cases of gestational MDR-TB and its prevalence is likely to increase because of increased resurgence of TB in pregnancy. Biological changes and relative immunocompromised status of pregnancy may allow latent infection to progress to active tuberculosis [5–8]. As teratogenic potential of second line drugs is also not clear, therefore effective contraception is strongly advocated for all nonpregnant women receiving drug-resistant treatment. Nonetheless, MDR-TB, if left untreated or undiagnosed in pregnancy, is associated with higher maternal morbidity, mortality, and increased risk of vertical transmission. There is increased risk of obstetrical complications like spontaneous abortions, fetal growth restriction, oligohydramnios, preterm labor, and increased neonatal mortality [8–11]. More assertive treatment with second line drugs is required for treating such cases which are generally more virulent and require closed supervision. Prolonged antitubercular treatment of 18–24 months after sputum culture conversion is the standard of care for such patients.

## 2. Case Reports

### 2.1. Case History I

A 24-year-old primigravida at 35-week period of gestation (POG) with MDR-TB was admitted in view of severe growth restriction and oligohydramnios. She had past history of pulmonary tuberculosis at the age of 14 years for which she received treatment for 6 months. She had relapse of pulmonary tuberculosis 1 year back and was suspected to have MDR-TB in view of worsening symptoms along with sputum positivity after 6 months of first line treatment. Sputum testing revealed drug resistance for isoniazid and rifampicin. The patient was started on second line antitubercular drugs, that is, ethionamide, cycloserine, levofloxacin, ethambutol, and pyrazinamide, along with injection of kanamycin for six months till sputum conversion. In continuation phase, injection of kanamycin was omitted and the patient continued to use the rest of the drugs. She conceived while on same treatment and was continued on the same drugs after consultation with pulmonologist. Her general physical examination did not reveal any abnormality and HIV and the rest of the routine antenatal investigations were normal. Fetal evaluation revealed severe growth restriction and oligohydramnios at 28 weeks of gestation. Fetal surveillance was continued with biweekly biophysical profile; she was given antenatal steroids and caesarean was performed at 35 weeks in view of breech with growth restriction with severe oligohydramnios. She delivered healthy baby with birth weight of 1.2 kg (standard deviation of less than 2) with Apgar score of 8 and 9 and there were no obvious congenital malformations. Neonatal work-up for tuberculosis revealed normal chest X-ray and Mantoux and gastric aspirate, cerebrospinal fluid analysis, and urine tested for acid fast bacilli were also negative. Placental membrane tested for acid fast bacilli was also negative. Baby was started on breast-feeding and given isoniazid prophylaxis. Infant's evaluations at 3 months were negative for tuberculosis and the baby continued on the same prophylaxis for 6 months.

### 2.2. Case History II

**Figure 1:**
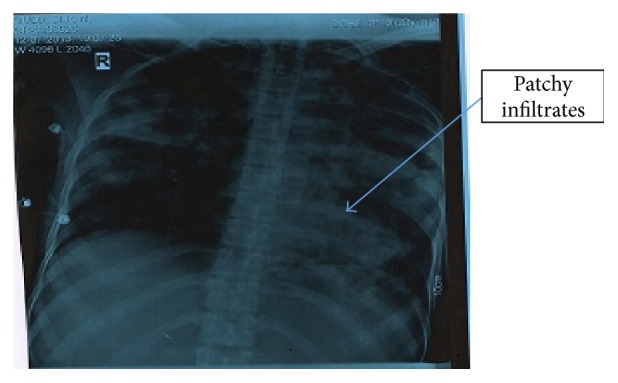


A 20-year-old primigravida was referred at 33 + week's period of gestation in view of severe intrauterine growth restriction with oligohydramnios. She has been on antitubercular treatment for past 5 months but was noncomplaint, continued to have fever and cough with expectoration, and did not have sputum conversion till date. She had infrequent antenatal checkups and obstetrical ultrasonography at 30 weeks revealed intrauterine growth restriction along with oligohydramnios. On admission, her general physical examination revealed fever, pallor, tachycardia, tachypnea, and coarse crepitations (auscultation) on bilateral chest. Chest X-ray showed cavitary lesions along with pneumonic patches (Figure 1) and sputum tested for drug resistance panel revealed drug resistance against rifampicin and isoniazid. She went into spontaneous preterm labor at 35 weeks and delivered a live-born baby boy of 1.5 kg with Apgar score of 8 and 9. Neonatal Mantoux and gastric aspirate were negative for acid fast bacilli. In view of sputum positivity of the mother, the baby was isolated from the mother and given expressed milk. BCG immunization was withheld and the baby was started on isoniazid prophylaxis. Placental histology was reported as normal with no tubercular involvement. Infantile evaluation at 3 months reveled no evidence of tubercular infection and the mother was started on second line drugs along with injection of kanamycin in postpartum period.

## 3. Discussion

Gestational MDR-TB needs the same consideration during treatment as is currently held related to the use of first line drugs during pregnancy. Paucity of data and lack of consensus regarding management of MDR-TB during pregnancy set it as controversial issue. Only case reports provide guidance for management of multidrug-resistant (MDR) tuberculosis in pregnancy [5, 6, 12, 13]. Most clinicians had discouraged patients from becoming pregnant or proceeding with existing pregnancy [14]. In many times these patients are undertreated by health care providers because of posed management challenges, that is, insufficiency of data regarding safety profile, restraining use of rifampicin and isoniazid, timing of commencement of treatment, compelling evidence of toxicity, and fear of posttreatment complications.

Management of multidrug-resistant tuberculosis (MDR-TB) in pregnancy is like a double-edged sword. On one hand, second line antitubercular drugs used for the treatment are potentially teratogenic, less effective, and more noxious; on the other hand, suboptimal treatment of such patients may be hazardous. Therefore, management of these patients involves multidisciplinary approach with the team comprising obstetrician, neonatologist, pulmonologist, and public health experts. Treatment regimens and duration of therapy for such patients need individualization in accordance with susceptibility pattern of infective strain. Therapy is usually delayed until second trimester in order to avoid teratogenic effect of the drugs unless patient is HIV positive or in critical condition [5].

Most of the second line drugs are under category C except aminoglycosides which are under category D (Table 1). There have been several case studies published; some have shown no adverse perinatal outcomes, while others have recorded growth restriction and congenital defects [6, 12, 13, 15]. In a retrospective case study of 38 Peruvian pregnant women treated for MDR tuberculosis, 61% of them were cured, 13% died, and 5% had experienced treatment failure. Eight (21%) women experienced pregnancy complications, such as spontaneous abortion and vaginal bleeding. No infants displayed teratogenic effects [6]. Among 5 South African pregnant women with MDR tuberculosis, 3 had HIV and 2 experienced adverse drug events (deafness and drug-induced hepatitis). The infants showed no evidence of teratogenicity [15].

Favored regimens for MDR-TB outside pregnancy are ethionamide, pyrazinamide, kanamycin, levofloxacin, ethambutol, and cycloserine during 6–9 months of the intensive phase and 4 drugs, levofloxacin, ethionamide, ethambutol, and cycloserine, during the 18–24 months of the continuation phase [16, 17]. However, few considerations and modifications are needed to use these drugs during pregnancy. Aminoglycosides are avoided in the regimens of pregnant patients because of fear of ototoxicity to the developing fetus. Although capreomycin is found to possess the risk of ototoxicity, it is the drug of choice, if injectable is unavoidable and the drug level in the fetus is minimized by using capreomycin thrice weekly from the beginning. Use of prothinamide and ethionamide is debatable in pregnancies as growth retardation, abortions, malformations, and CNS defects are documented in animal studies; therefore they are usually replaced with para-aminosalicylic acid (PAS). Safety of PAS has been questioned in the past but the results were not convincing and now it is considered as one of the safest second line drugs [18]. Fluoroquinolone use during pregnancy does not seem to increase the risk of malformations as concluded in meta-analysis by Bar-Oz et al. [19]; so, despite limited data on the safety and long-term use of fluoroquinolone, cycloserine, PAS, and amoxicillin/clavulanate in pregnancy, they are considered the drugs of choice for M/XDR-TB treatment during pregnancy. Index case 1 received ethionamide throughout her pregnancy but no malformation was seen in the baby. This is in agreement with observation by Schardein in many case reports [5, 20]; nonetheless, many investigators still disfavor its use. Similar to many case series of MDR-TB in pregnancy, obstetrical complications like oligohydramnios, intrauterine growth restriction, and preterm labor were seen in our both patients [5, 6, 8, 12]. Despite these documented complications careful and appropriate decision of treatment initiation resulted in clinical cure of majority of pregnant women with improved neonatal outcome. As there is limited data on the safety of delamanid and bedaquiline in pregnancy for the treatment of M/XDR-TB, these drugs should be avoided.

Breast-feeding is as such not contraindicated if patient had sputum conversion because only a meager fraction of therapeutic dose of drugs is excreted in milk [20] and any effect on infants of such exposure during the full course of MDR-TB treatment has not been established. However, if mother is sputum smear-positive, the care of the infant should be taken by family members until she becomes sputum smear-negative, if this is feasible. There are different recommended policies across the world regarding separation of the child from the mother and the use of top milk [21]. In case of good resource settings, provision of infant formula can be an alternative to breast-feeding, but evidence supporting this fact is weak. Because of immunological benefits, efforts should be taken to support breast-feeding and expressed breast milk feeding can be used as a substitute, with personal hygiene when there are mothers who have MDR-TB or are sputum smear-positive during delivery. In the first case, the infant was started on breast-feeding because the mother was sputum-negative; however, the neonate was separated from his mother in the second case because the mother was sputum-positive. True congenital tuberculosis is extremely rare and risk is more shortly after birth. The child should receive Bacillus Calmette–Guérin (BCG) vaccination at birth as per WHO policy, and the role of INH prophylaxis in neonates born to mothers with MDR-TB is not clear. In our scenario we did give INH prophylaxis to both babies.

On the basis of available literature, we conclude that although pregnancy complicates the management of MDR-TB, the known and theoretical benefits of continuing treatment seem to outweigh theoretical risks to the mother and fetus. Cautious assessment of pregnant patients taking into consideration the period of gestation and severity of disease is required before initiation of the treatment with primary goal of sputum conversion before delivery.

## Figures and Tables

**Table 1:** Safety of MDR-TB medicines during pregnancy.

Medication	Safety class	Comments
Ethambutol	B	Experience in gravid patients suggests safety.
Pyrazinamide	C	Use with caution. Most references suggest it is safe to use.
Aminoglycosides^a^		
Streptomycin	D	Avoid use. Documented toxicity to developing foetal ear. Risks and benefits must be carefully considered. Avoid use when possible.
Kanamycin	D	
Amikacin	D	
Capreomycin	C	
Fluoroquinolones		
Levofloxacin	C	Use with caution. No teratogenic effects seen in humans when used for short periods of time (2–4 weeks). Associated with permanent damage to cartilage in weight-bearing joints of immature animals. Experience with long-term use in gravid patients is limited, but, given bactericidal activity, benefits may outweigh risks.
Moxifloxacin	C	
Gatifloxacin	C	
Ethionamide/prothinamide^a^	C	Avoid use. Teratogenic effects observed in animal studies; significantly worsens nausea associated with pregnancy.
PAS	C	Causes fatal diarrhea.
Cycloserine/terizidone	C	Significant experience in gravis patients: animal studies have documented toxicity.
Amoxicillin/clavulanic acid	B	Compatible with breast-feeding. Causes fetal necrotizing enterocolitis.
Rifabutin^a^	B	Unknown compatibility with breast-feeding.
Linezolid	C	Not yet approved for tuberculosis treatment; reduced efficacy with rifampicin.

^a^Decreases efficacy of BCG vaccine.
